# From Hiring to Firing: Activation of Inhibitory Neurons and Their Recruitment in Behavior

**DOI:** 10.3389/fnmol.2019.00168

**Published:** 2019-07-03

**Authors:** Olivia K. Swanson, Arianna Maffei

**Affiliations:** ^1^Department of Neurobiology and Behavior, SUNY—Stony Brook, Stony Brook, NY, United States; ^2^Graduate Program in Neuroscience, SUNY—Stony Brook, Stony Brook, NY, United States

**Keywords:** GABA, synapses, circuit, excitability, plasticity

## Abstract

The investigation of GABAergic inhibitory circuits has substantially expanded over the past few years. The development of new tools and technology has allowed investigators to classify many diverse groups of inhibitory neurons by several delineating factors: these include their connectivity motifs, expression of specific molecular markers, receptor diversity, and ultimately their role in brain function. Despite this progress, however, there is still limited understanding of how GABAergic neurons are recruited by their input and how their activity is modulated by behavioral states. This limitation is primarily due to the fact that studies of GABAergic inhibition are mainly geared toward determining how, once activated, inhibitory circuits regulate the activity of excitatory neurons. In this review article, we will outline recent work investigating the anatomical and physiological properties of inputs that activate cortical GABAergic neurons, and discuss how these inhibitory cells are differentially recruited during behavior.

## Introduction

Inhibitory interneurons constitute a small but crucial neuronal class in the cortex. While these cells comprise only 10%–20% of the total neural population, their connectivity and recruitment are essential in sensation, movement, and cognition. One difficulty in synthesizing the role of inhibitory cells lies in their diversity: these neurons express an array of molecular markers and have heterogeneous firing properties as well as distinct synaptic connectivity (Kubota, 2014). However, the diversity of inhibitory neurons allows these cells to provide the appropriate inhibition for a wide variety of stimuli and behaviors. Great strides have been made in identifying clusters of inhibitory interneuron groups based on their varying gene expression (Cauli et al., 2000; Kubota et al., 2011; Tasic et al., 2016; Paul et al., 2017). These data demonstrate that while some genes are expressed to varying degrees across several interneuron types, there are certain non-overlapping markers that can be used to delineate broad groups of inhibitory interneuron groups. This review article will focus on three largely non-overlapping classes of inhibitory interneurons in the rodent cortex that express the following molecular markers: parvalbumin (PV^+^), somatostatin (SST^+^), and type 3 serotonin receptor (5HT_3_), particularly focused on 5HT_3_^+^ neurons that express vasoactive intestinal peptide (VIP^+^; Xu et al., 2010; Rudy et al., 2011; Tremblay et al., 2016). PV^+^ inhibitory neurons are typically fast-spiking basket cells, found mainly in layers 4 and 5, that preferentially contact the perisomatic region of pyramidal neurons (Nassar et al., 2015; Neske et al., 2015). SST^+^ inhibitory neurons include Martinotti cells found in layers 5 and 6 that contact dendrites of pyramidal neurons (Yavorska and Wehr, 2016). VIP^+^ interneurons are bipolar or multipolar inhibitory neurons, found most densely in layer 2/3, that exert disinhibitory control in the cortex by synapsing onto other inhibitory neuron groups (Pronneke et al., 2015). These inhibitory neurons also have a high level of interconnectivity, with each subtype displaying a connection preference to one another, as well as neighboring pyramidal neurons (Jiang et al., 2015; Tremblay et al., 2016). Taken together, inhibitory interneuron classes span all layers of the cortical mantle and thus can powerfully regulate excitatory activity across the cortex. As inhibition is an essential mechanism in maintaining balanced cortical activity (Isaacson and Scanziani, 2011; Tatti et al., 2017), long-range inputs to a given cortical area often simultaneously activate one or more types of inhibitory interneurons as well as pyramidal neurons. This review article will discuss recent results regarding the recruitment of GABAergic neurons by long-range driving and modulating inputs. We will then discuss how the recruitment of cortical interneurons plays a role in the function of cognitive, motor, and sensory cortices.

## Thalamic Excitation of Inhibitory Neurons

A major source of excitation to cortical inhibitory neurons arises from the thalamus. Generally, GABAergic neurons receive the largest input from thalamic regions most functionally relevant to their own cortical region, and excitation *via* these pathways is not uniform across interneuron subtype. For example, anatomical studies indicate that PV^+^, SST^+^, and VIP^+^ neurons in somatosensory cortex (S1) receive similar innervation from the ventroposteromedial (VPM) and the posteromedial nucleus (POm) of the thalamus, which are two major thalamic inputs to S1 that are widely known to transmit somatosensory-related signals to the cortex (Landisman and Connors, 2007; Castejon et al., 2016; Wall et al., 2016). However, electrophysiological studies in S1 reveal that thalamocortical (TC) inputs onto these neurons are not congruent: PV^+^ neurons respond with a higher connection probability, higher likelihood to spike, and strong synaptic depression to subsequent stimulation, while SST^+^ neurons show lower connection probability and facilitating, smaller magnitude responses that have a longer latency from stimulus onset (Cruikshank et al., 2007, 2010; Tan et al., 2008). Additionally, the response of PV^+^ interneurons is often comparable to or larger than that of a simultaneously recorded excitatory neurons (Cruikshank et al., 2007, 2010), and they can mediate powerful feedforward inhibition following TC stimulation, particularly in layer 4 (Sun et al., 2006). Input from higher-order thalamic nuclei, like the POm, activates PV^+^ and VIP^+^ interneurons but suppresses SST^+^ neurons (Audette et al., 2018; Williams and Holtmaat, 2019). Inputs to inhibitory neurons from the POm also show laminar specificity, with PV^+^ interneurons showing highest connection probability and response amplitude in layer 5 and inhibitory neurons expressing 5HT_3_, including VIP^+^ cells, showing largest amplitudes in superficial layers. As VIP^+^ interneurons tend to have a disinhibitory action on cortical circuits, their activation by a high order somatosensory thalamic projection may play a role in the recently reported powerful, long-lasting excitation of superficial S1 by the POm (Zhang and Bruno, 2019).

In the primary auditory and visual cortices, TC input (from the medial geniculate body and the lateral geniculate nucleus, respectively), drives excitatory postsynaptic currents (EPSCs) in PV^+^, SST^+^ and VIP^+^ interneurons, however, PV^+^ interneurons have a higher connection probability with TC axons, and the input is larger than that of the other interneurons, as well as excitatory neurons (Kloc and Maffei, 2014). In both cortices, TC input to SST^+^ and VIP^+^ interneurons is largely restricted to layer 4, where these neurons show a low connection probability to this input and the magnitude of current is 1/10 of that onto PV^+^ cells (Ji et al., 2016). Similar results were reported in frontal, cognitive-associated cortices: Electron microscopy studies showed that axons from the mediodorsal thalamus synapse onto at least three types of inhibitory neurons, including PV^+^, calretinin^+^, and calbindin^+^ (Rotaru et al., 2005). Calretinin and calbindin are calcium-binding proteins used to mark interneurons, and each has been shown to colocalize to a considerable degree with VIP and SST, respectively (Gonchar et al., 2007). Despite anatomically defined inputs onto each of these inhibitory neurons, electrophysiological stimulation of this pathway revealed that the mediodorsal thalamus drives feedforward inhibition *via* PV^+^ but not SST^+^ interneurons (Delevich et al., 2015). An electron microscopy study in the secondary motor cortex (M2) has also shown that motor thalamic input from the ventroanterior and ventromedial nuclei made synapses onto the soma and dendrites of PV^+^ interneurons in L2/3 and L5, respectively (Shigematsu et al., 2016). Finally, in the presubiculum, which is a region in the parahippocampal cortex involved in spatial orientation of the head, the anterior thalamic nuclei carrying head direction-related information synapse onto PV^+^ but not SST^+^ neurons (Nassar et al., 2018). Taken together, these data suggest that TC pathways synapse onto a variety of cortical inhibitory cells, including those expressing PV, SST, and VIP. While anatomical tract tracing studies confirm that TC axons form synapses onto these interneuron subtypes, electrophysiological analyses of these inputs reveal that PV^+^ neurons are the most commonly targeted subtype, and they also receive the strongest input. This could possibly be explained by a differential somatodendritic localization of TC synapses onto each inhibitory neuron type. Several studies have investigated the distribution of TC boutons along PV^+^ neurons in S1 (Bagnall et al., 2011; Kameda et al., 2012; Hioki, 2015). These studies revealed that TC inputs to PV^+^ neurons can show differential anatomical organization that correlates with the power of the connection, where the strongest synaptic input was provided by a concentrated cluster of release sits on the primary dendrites of the GABAergic cell. In contrast, a similar study focused on VIP^+^ interneurons showed that these cells mainly receive thalamic input along their distal dendrites (Sohn et al., 2016). Further studies connecting the anatomic location of TC synapses with physiology data would possibly bridge synapse location and response strength for each inhibitory cell type. For example, in primary visual cortex (V1), TC input to fast-spiking interneurons in the V1 is due to the activation of several powerful release sites (Kloc and Maffei, 2014), but whether the structure/function relationship at these synapses follows this motif, and is generalized to all TC inputs, is unknown. A thorough understanding of the location of all TC synapses on each inhibitory neuron subtype is essential to synthesize these bodies of data.

## Corticocortical Excitation of Inhibitory Neurons

Inhibitory neurons are also excited by inter-areal cortical inputs. While less is known about the anatomy and physiology of long-range cortical inputs onto GABAergic neurons, there have been several interesting trans-synaptic tracing studies of these pathways that suggest that these cells receive a highly diverse set of inputs from many cortical areas. As expected, the source of these cortical inputs depends on the function of the cortex studied: for example, GABAergic neurons in the barrel cortex are contacted by axons from cortical areas including the ipsilateral secondary somatosensory cortex, the contralateral S1, and the primary motor cortex (M1), while the inputs from other sensory or limbic cortices is limited (Wall et al., 2016). When analyzed on an anatomical level, the proportion of input from these cortical sources onto PV^+^, SST^+^, and VIP^+^ neurons was comparable. Optogenetic stimulation of the corticocortical pathway from M1 to S1, however, revealed that this input is strongest onto VIP^+^ interneurons (Lee et al., 2013). VIP^+^ interneurons showed the largest response to M1 input which exceeded that of simultaneously recorded pyramidal neurons, and these responses showed synaptic depression. PV^+^ interneurons also showed depressing responses comparable to that of pyramidal neurons, while SST^+^ interneurons had the weakest connection, with facilitating responses that were much smaller than those of excitatory cells. In the visual system, trans-synaptic tracing performed in V1 showed that PV^+^ neurons receive input from the secondary visual cortex, auditory cortex (A1), S1, parietal association area, M2, and the contralateral V1 (Lu et al., 2014). Functional study of this excitatory input to V1 PV^+^ neurons from M2 and contralateral V1 has revealed that these inputs exhibit strong short-term depression. However just as there is a high level of variety in corticocortical (CC) projections, there is also diversity in the postsynaptic targets of these pathways. For example, the cingulate cortex projects to the ipsilateral V1, and selective inactivation of either PV^+^, SST^+^, or VIP^+^ neurons coinciding with this pathway during a visual discrimination task disrupted normal center-surround modulation (Zhang et al., 2014). This suggests that there are specific motifs for inhibitory interneuron activation dependent on the CC pathway.

A common feature of CC activation of inhibitory neurons is the generation of feedforward inhibition, mediated largely by PV^+^ interneurons. In the prefrontal cortex, both PV^+^ and SST^+^ interneurons receive a monosynaptic, glutamatergic input from the contralateral cortex (Anastasiades et al., 2018). While activation of this pathway can drive both PV^+^ and SST^+^ neurons to fire, a suprathreshold response is more frequent in PV^+^ interneurons, which could indicate they are the primary drivers of feedforward inhibition in this pathway. Feedforward inhibition by PV^+^ interneurons was also observed in the callosal input to A1: inputs from the contralateral A1 make synapses onto PV^+^ and SST^+^ interneurons, and this callosal activation of PV^+^ neurons drives selective inhibition of adjacent CC-projecting pyramidal neurons (Rock and Apicella, 2015; Oviedo, 2017). However, this does not appear to be the case regarding callosal input to motor cortices, at least not in deep layers: electron microscopy of these inputs to layer 6 PV^+^ interneurons show a direct synaptic connection, however, feedforward inhibition can only be evoked following callosal stimulation in a subset of neighboring pyramidal neurons (Karayannis et al., 2007). This suggests that feedforward inhibition following callosal stimulation may be limited to specific cortical layers or regions.

## Other Sources of Excitation

In addition to thalamic and CC inputs, GABAergic interneurons in cortical circuits can be recruited by the amygdala. While the amygdala is known for processing signals related to emotions and fear memory, the recruitment of cortical inhibitory circuits by amygdalar projections remained controversial until recently. Publications from several groups reported that amygdalocortical pathway activation can have both excitatory and inhibitory effects, suggesting that perhaps the amygdala engages both excitatory and inhibitory circuits in the cortex (Yamamoto et al., 1984; Hanamori, 2009). A recent study demonstrated that the basolateral nucleus of the amygdala (BLA) can evoke feedforward excitatory and inhibitory responses in the insular cortex (Stone et al., 2011), further bolstering this idea. These results were confirmed and expanded by studies examining amygdalocortical projections to a variety of cortical circuits: in the prefrontal cortex, the BLA projects directly onto PV^+^ GABAergic neurons, which in turn exert feedforward inhibition onto nearby pyramidal cells (Dilgen et al., 2013; Cheriyan et al., 2016). Subsequent studies using optogenetic approaches to selectively stimulate BLA afferents demonstrated that BLA axons make synapses onto both PV^+^ and SST^+^ interneurons in the insular and prefrontal cortex (Haley et al., 2016; McGarry and Carter, 2016). This input is robust onto both interneuron types, however, analysis of this synapse’s short-term dynamics revealed that BLA input to PV^+^ interneurons is depressing, while input onto SST^+^ interneurons is stable or facilitating across trains of stimuli. This suggests that excitatory inputs from the BLA to cortical GABAergic interneurons follow the same short-term dynamics as those from thalamic and cortical sources.

## Modulation of Inhibitory Neurons

In addition to being directly recruited by glutamatergic inputs, inhibitory neurons are known to express receptors for neuromodulators, indicating that their activity is also subject to state changes and the release of a variety of neurotransmitters. Interestingly, while all three inhibitory neuron types express neuromodulatory receptors, the ratio of expression is unique to each (Paul et al., 2017). For example, PV^+^ neurons preferentially express the genes for serotonin and opioid receptors, while SST^+^ cells express a wider variety of neuromodulatory receptor genes. Results showed that SST^+^ interneurons express genes for cholinergic, serotonergic, and oxytocinergic receptors, as well as those that bind substance P and orexin. VIP^+^ interneurons showed the highest and most diverse expression for neuromodulatory receptors, including those that bind serotonin, acetylcholine, neuropeptide Y, and catecholamines. Another study found that PV^+^ and SST^+^ inhibitory neurons in the prefrontal cortex express neurotensin-1 receptors that are activated by neurotensin co-released by dopaminergic afferents in this cortical area (Petrie et al., 2005). Release of neurotensin within the prefrontal cortex increased extracellular GABA, indicating that neuromodulation of these interneurons can directly lead to changes in inhibitory activity.

PV^+^, SST^+^, and VIP^+^ neurons in the barrel cortex receive input from the Basal nucleus of Meynert, which is a source of cholinergic input (Wall et al., 2016). This anatomical work is further supported by transcriptional analysis in M1 and S1 showing that all three GABAergic interneuron subtypes express cholinergic receptors (Paul et al., 2017). Cholinergic modulation of inhibitory interneurons has also been observed in V1, where stimulation of the pathway from the Basal nucleus of Meynert to V1 decorrelates neural response *via* SST^+^ interneuron activity (Chen N. et al., 2015). In V1, an *in vivo* calcium imaging study showed that stimulation cholinergic input to the cortex modified the responses of nearly all VIP^+^ interneurons studied, and roughly half of PV^+^ neurons, while SST^+^ interneurons were rarely affected (Alitto and Dan, 2012). Interestingly, cholinergic stimulation consistently increased intracellular calcium in VIP^+^ interneurons, while in PV^+^ interneurons the responses were heterogeneous.

The noradrenergic system also differentially engages GABAergic neurons. Stimulation of the locus coeruleus, which provides the source of noradrenaline to the cortex, drives an increase in cFos expression in PV^+^ and SST^+^ and, to a lesser extent, VIP^+^ neurons (Toussay et al., 2013). In the rat frontal cortex, noradrenaline depolarizes fast-spiking (putative PV^+^) interneurons, while it depolarizes and drives SST^+^ interneurons to fire (Kawaguchi and Shindou, 1998).

**Figure 1:**
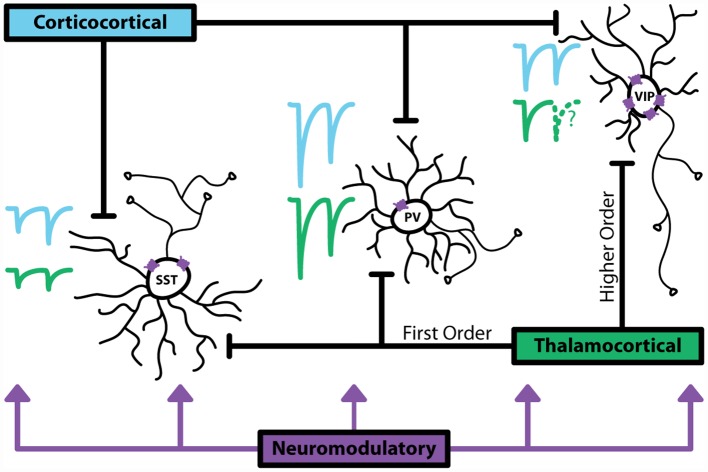
Major sources of activation to inhibitory interneurons. Corticocortical (CC) inputs are represented with blue traces, thalamocortical (TC) inputs are represented with green traces. Relative sizes of excitatory postsynaptic currents (EPSCs) show that generally parvalbumin (PV^+^) interneurons receive large, depressing inputs. Somatostatin (SST^+^) interneurons receive smaller inputs that facilitate. vasoactive intestinal peptide (VIP^+^) interneurons have been shown in anatomical studies to receive synapses from CC and TC pathways, however, data characterizing the magnitude and dynamics of these synapses is limited. VIP^+^ express the highest, more diverse levels of neuromodulatory receptors, indicating that these interneurons are a major target for non-glutamatergic or GABAergic activation. Dotted lines indicate lack of data.

Together, these studies highlight the complexity of the recruitment structure for inhibitory interneurons (Figure 1). These cells are poised at a critical position within cortical circuits: they are often activated by long-range glutamatergic and modulatory inputs alongside excitatory neurons, and thus can act as gating mechanisms for cortical activity. The diversity of means to drive inhibitory neurons also indicates that the function of GABAergic cells goes well beyond simply controlling principal neuron excitability.

## Inhibitory Neurons and Cognition

The recent availability of a variety of experimental tools for the selective activation/inactivation of GABAergic neurons facilitated the investigation of their contribution to complex functions. GABAergic neuron activity in the prefrontal cortex is necessary for several aspects of healthy cognition. Using a selective toxin, PV^+^ neuron-specific disruption produced cognitive deficits comparable to those observed following non-specific prefrontal cortex lesion (Murray et al., 2015). Additionally, there is a small population of PV^+^ and VIP^+^ neurons in the prefrontal cortex that project to the nucleus accumbens (Lee et al., 2014). Stimulation of this long-range GABAergic pathway induced avoidance behavior while the animal performed a place preference task, suggesting that these neurons are involved in conveying aversive signals to the accumbens. Inhibitory activity in the anterior cingulate cortex is also engaged during foraging tasks, with inhibitory neuron subtypes engaged differentially during specific aspects of the behavior (Kvitsiani et al., 2013). PV^+^ interneurons were most active when animals were leaving the reward zone, while SST^+^ interneurons were highly active until animals entered the reward zone. Subtype-specific recruitment of inhibitory neurons has also been observed during working memory tasks (Kim et al., 2016). SST^+^ interneurons showed strong delay period target-dependent activity and only narrow-spiking SST^+^ cells were suppressed by reward. Differently, PV^+^ interneurons did not show strong activity during the delay period, however, nearly all were strongly suppressed by reward. It is also important to note that these interneurons have been implicated in the generation of synchronized neural firing, specifically that of gamma and theta oscillations (Fanselow et al., 2008; Gonzalez-Burgos and Lewis, 2008; Sohal et al., 2009). In the cortex, PV^+^ interneurons appear to be involved in gamma oscillations, while SST^+^ interneurons play a role in theta oscillations. Synchrony between brain areas is important for working memory, memory retrieval, cognitive integration, and information processing, thus the proper activity of inhibitory interneurons is integral to the generation of specific brain states and healthy cognition.

How these groups of neurons are recruited by their inputs during executive functions and the mechanisms regulating their responses are still under investigation. Compared to sensory cortices, the activity of specific inhibitory neuron populations in prefrontal areas remains understudied. To fully understand the role of these cells in cognition, it will require a synergistic approach relying on experimental and theoretical efforts that examine these processes at varied levels of resolution to bridge the gap between connectivity and functional recruitment.

## Inhibitory Neurons and Motor Function

**Figure 2:**
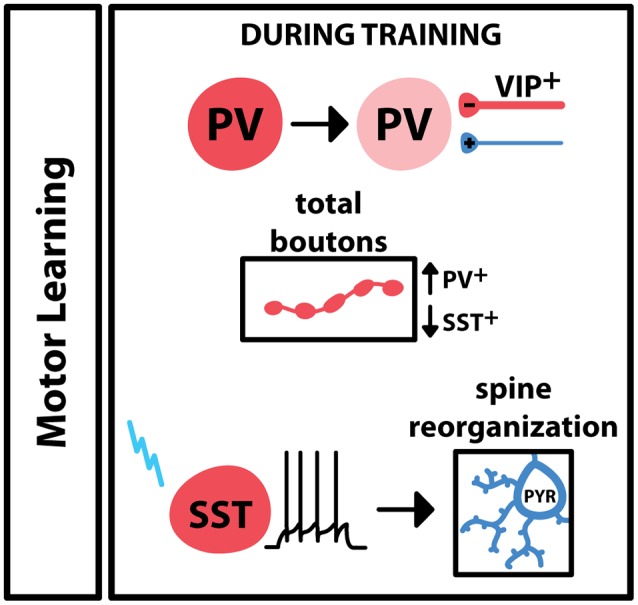
Engagement of GABAergic interneurons during motor learning. In primary motor cortex (M1), motor training drives a decrease in the expression of PV in PV^+^ interneurons, and an increase of VIP^+^ boutons onto these cells. Overall PV^+^ boutons increase during training; SST^+^ boutons decrease during training. Motor learning is associated with changes in pyramidal spine dynamics, and these changes have been shown to follow specific activation of the SST^+^ interneuron.

Motor learning leads to the engagement of inhibitory elements in M1, which leads to plastic changes at both excitatory and inhibitory synapses (Figure 2). 2-photon imaging of axon terminal fields in M1 reported an increase in PV^+^ boutons during training of a lever-press task in mice, while the training of this task led to a decrease in SST^+^ boutons (Chen S. X. et al., 2015). Interestingly, activation or deactivation of SST^+^ neurons led to bidirectional changes in spines on neighboring pyramidal neurons. Pyramidal neuron spine reorganization is a common feature of learning, so these results suggest that during motor training activity of SST^+^ interneurons is important for learning-dependent plasticity. Additionally, animals trained to run on an accelerating rotarod show a switch in PV expression in M1, as well as inhibitory input onto these cells, across the learning period: during the training period, there is low PV expression in the during training period, with an increase in VIP^+^ inhibitory boutons onto PV^+^ cells (Donato et al., 2013). Once performance saturates, PV expression in M1 is high, accompanied by a reduction in inhibitory boutons and an increase in excitatory boutons onto these PV^+^ neurons. Taken together, these results indicate that PV^+^, SST^+^ and VIP^+^ inhibitory interneurons are differentially engaged during motor learning. GABAergic interneurons are also engaged during the execution of a learned movement: one study trained mice on a sensory stimulation-triggered reaching task and used extracellular recordings and optotagging to selectively monitor regular spiking and fast-spiking cell populations (Estebanez et al., 2017). The results of this study showed that PV^+^ interneurons in M1 increased their firing in response to the sensory cue as well as the onset of reaching, suggesting that PV^+^ interneurons additionally participate in voluntary movement execution. Another study focused on the role of a small group of PV^+^ and SST^+^ neurons that project from M1 and M2 to the dorsolateral striatum (Melzer et al., 2017). Transgenic cre-expressing animals and a floxed channelrhodopsin-expressing virus were used to selectively stimulate the axons of these cells within the striatum during spontaneous locomotion. When axons from SST^+^-M2 or PV^+^-M1 neurons were activated, locomotion decreased, while activation of M1-originating SST^+^ neuron axons increased locomotion. Overall, these data point to several different roles for inhibitory neurons in voluntary skilled movement. Specific populations of GABAergic neurons, even within subtypes, differ in activity and recruitment at specific phases or motor activity.

## Inhibitory Neurons and Sensory Processing

Subtype-specific recruitment of inhibitory interneurons for sensation and perception has been observed in several sensory cortices. In V1, recruitment of each inhibitory interneuron subtype has distinct effects on visual processing. Selective activation of PV^+^ interneurons using channelrhodopsin expressed exclusively in PV^+^ cells resulted in narrowed orientation tuning and increased direction selectivity in neighboring neurons (Lee et al., 2012). Activation of SST^+^ or VIP^+^ neurons did not recapitulate this effect, suggesting that it is mediated by PV^+^ neurons. SST^+^ interneurons, on the other hand, have been implicated in sensory integration and gating cross-modal signals reaching V1 (Scheyltjens et al., 2018). Finally, recruitment of VIP^+^ and SST^+^ neurons *via* a CC pathway from the cingulate cortex to V1 plays a role in center-surround modulation (Zhang et al., 2014). Behavioral states can also modulate the gain of excitatory neurons. Locomotion, in particular, increases the gain of V1 neurons with no effect on their spontaneous activity or tuning properties (Niell and Stryker, 2010). *In vivo* calcium imaging during locomotion and visual stimulation has shown that the activity VIP^+^ interneurons increases during locomotion, leading to an augmented response to visual stimuli in non-VIP^+^ neurons (Fu et al., 2014). SST^+^ interneurons consistently showed suppression of activity during locomotion, while PV^+^ interneurons had heterogeneous responses, consistent with the strong inhibitory connection between VIP^+^ and SST^+^ interneurons (Pfeffer et al., 2013). Furthermore, pharmacological blockade of nicotinic acetylcholine receptors attenuated the locomotion-induced response of VIP^+^ neurons, suggesting a functional role for subtype-specific cholinergic modulation of inhibitory neurons in V1 (Alitto and Dan, 2012). The VIP-SST inhibitory circuit is necessary for cortical plasticity in adults after a change in the level of visual stimulus: either activating VIP^+^ interneurons, or silencing SST^+^ neurons, is sufficient to increase visual cortical plasticity (Fu et al., 2015). Response features of VIP^+^ neurons are distinct depending on cortical area: in V1 they behave similarly to PV^+^ neurons in that they are broadly tuned to stimulation, while in A1, VIP^+^ interneurons behave unlike PV^+^ interneurons or pyramidal neurons, with a strong selectivity to sound intensity (Mesik et al., 2015). In A1, context switching from passive tone perception to active tone perception in a decision-based task differentially modulates GABAergic neurons by type (Kuchibhotla et al., 2017). VIP^+^ interneurons show the largest change in activity following the context switch, and they show the highest level of activity during the passive perception period. In contrast, PV^+^ and SST^+^ neurons increase their activity from the passive to active context.

## Interneurons and Sensorimotor Integration

**Figure 3:**
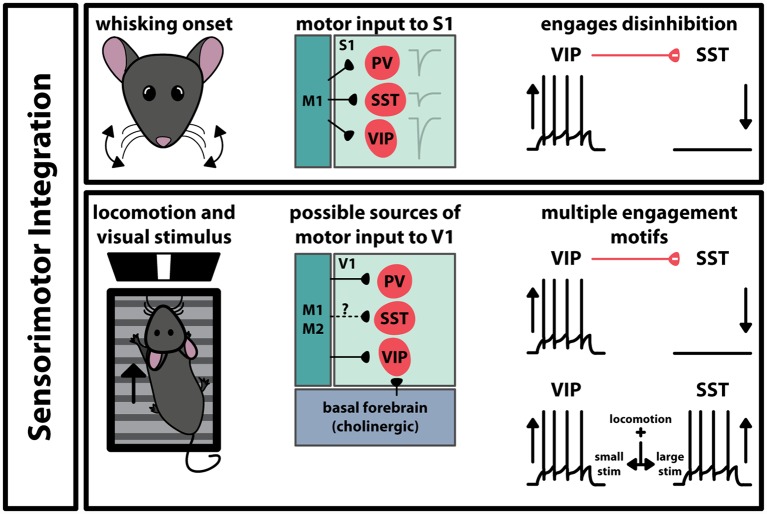
Engagement of GABAergic interneurons during sensorimotor integration. In somatosensory cortex (S1) and primary visual cortex (V1), movement during sensation activates specific pathways that differentially engage interneurons in the respective region. The paths transmitting movement-related signals to these areas differ, however, the coincidence of movement and sensation engages the VIP-SST disinhibitory circuit in both cases. In V1, the type of visual stimulus can influence which interneuron subtype alters its spiking activity.

Interneurons also play a role in the interaction between functionally connected brain regions. Movement is intrinsic to many sensory processes, so it stands to reason that there are pathways between motor and sensory cortices (Figure 3). For example, M1 projects to S1, making synaptic contact with all three interneuron subtypes (Kinnischtzke et al., 2016; Wall et al., 2016). This input is strongest onto VIP^+^ interneurons, which in turn inhibit SST^+^ interneurons (Lee et al., 2013). Interestingly, VIP^+^ interneurons increase their spiking probability during active whisking, while SST^+^ interneurons decrease their activity. Acute inactivation of vibrissal M1 with tetrodotoxin had no overall effect on local field potentials in S1, however, it did significantly reduce the correlation between whisking and increase VIP^+^ interneuron activity. This suggests that motor input to S1 engages a disinhibitory circuit that involves the activation of VIP^+^ interneurons and the suppression of SST^+^ interneurons. A similar circuit has been observed in V1: PV^+^, SST^+^, and VIP^+^ interneurons have been shown to receive input from M2 and to a lesser extent M1 (Lu et al., 2014; Leinweber et al., 2017). Locomotion has been shown to enhance visual perception, and studies have shown that GABAergic cells in V1 modulate their activity during locomotion while animals experience visual stimulus (Figure 3). In one study, VIP^+^ interneurons increase their activity during locomotion, and SST^+^ interneurons decreased activity (Fu et al., 2014). The increase in VIP^+^ interneuron activity is tied to nicotinic acetylcholine receptor activity, activated by an input from the basal forebrain. However, it is important to note that these results are controversial. Subsequent studies have shown that GABAergic neuron modulation depends on the context and magnitude of the visual stimulus and that this disinhibitory circuit model may not be the only way that locomotion can engage interneurons in V1 (Pakan et al., 2016; Dipoppa et al., 2018). These results suggest that even within a specific function, the brain may employ different interneuron subtypes depending on the characteristics of the sensory stimulus.

## Conclusions

While there are commonalities in the engagement of GABAergic cells across sensory regions, these data suggest that the activation of inhibitory interneurons is customized to the function of the region in which they reside. There have been great advancements in the development of tools for the investigation of inhibitory circuits and for the identification of specific GABAergic neuron groups. Anatomical and *in vitro* studies have made strides in elucidating the sources of activation for these cells, demonstrating that these cells receive diverse synaptic input from many thalamic, cortical, amygdalar, and neuromodulatory regions. Additionally, studies performed in behaving animals have shed light on the active roles these interneurons play in sensation, cognition, and movement. The next step in understanding the full picture of inhibitory function is to determine how the recruitment structure of these cells is used to drive inhibition during behavior. Synthesizing common roles of inhibitory cells across areas is difficult, in part because the behavioral paradigms used to engage one cortex may be difficult to compare to a task used in another region. Furthermore, while we did not extensively discuss GABAergic plasticity in this review article, it is well known that inhibitory circuits are dynamically regulated, and the efficacy of inhibitory synaptic transmission is activity-dependent. Thus, the functional engagement of cortical GABAergic neurons can powerfully expand the computational capacity of other neuron types and the neural network. We have examined the overarching similarities of inhibitory circuits across cortical regions while pointing out that some properties of these cells may be tailored to the area’s specific function. Expanding our knowledge of how each inhibitory neuron is recruited and the role that they play in shaping behavioral output remains a fundamental step to understand how the brain functions in health and disease.

## Author Contributions

Both authors were involved in writing and commenting the manuscript.

## Conflict of Interest Statement

The authors declare that the research was conducted in the absence of any commercial or financial relationships that could be construed as a potential conflict of interest.
